# Demonstration of disinfection procedure for the development of accurate blood glucose meters in accordance with ISO 15197:2013

**DOI:** 10.1371/journal.pone.0180617

**Published:** 2017-07-06

**Authors:** Shu-Ping Lin, Wen-Ye Lin, Jung-Tzu Chang, Chun-Feng Chu

**Affiliations:** 1 Graduate Institute of Biomedical Engineering, National Chung Hsing University, Taichung, Taiwan R.O.C; 2 Research Center for Sustainable Energy and Nanotechnology, National Chung Hsing University, Taichung, Taiwan R.O.C; 3 Jen-Ai Hospital, Taichung, Taiwan R.O.C; 4 Biotest Medical Corporation, Taichung, Taiwan R.O.C; University of California at Davis, UNITED STATES

## Abstract

Despite measures to reduce disease transmission, a risk can occur when blood glucose meters (BGMs) are used on multiple individuals or by caregivers assisting a patient. The laboratory and in-clinic performance of a BGM system before and after disinfection should be demonstrated to guarantee accurate readings and reliable control of blood glucose (BG) for patients. In this study, an effective disinfection procedure, conducting wiping 10 times to assure a one minute contact time of the disinfectant on contaminated surface, was first demonstrated using test samples of the meter housing materials, including acrylonitrile butadiene styrene (ABS), polymethyl methacrylate (PMMA), and polycarbonate (PC), in accordance with ISO 15197:2013. After bench studies comprising 10,000 disinfection cycles, the elemental compositions of the disinfected ABS, PMMA, and PC samples were almost the same as in the original samples, as indicated by electron spectroscopy for chemical analysis. Subsequently, the validated disinfection procedure was then directly applied to disinfect 5 commercial BGM systems composed of ABS, PMMA, or PC to observe the effect of the validated disinfection procedure on meter accuracy. The results of HBsAg values after treatment with HBV sera and disinfectant wipes for each material were less than the LoD of each material of 0.020 IU/mL. Before and after the multiple disinfection cycles, 900 of 900 samples (100%) were within the system accuracy requirements of ISO 15197:2013. All of the systems showed high performance before and after the series of disinfection cycles and met the ISO 15197:2013 requirements. In addition, our results demonstrated multiple cleaning and disinfection cycles that represented normal use over the lifetime of a meter of 3–5 years. Our validated cleaning and disinfection procedure can be directly applied to other registered disinfectants for cleaning commercial BGM products in the future.

## Introduction

Incidents of hepatitis B virus (HBV) transmission attributed to contaminated blood glucose meters (BGMs) have been reported [1–6]. It is strongly recommended that a BGM should not be shared and the user should perform a self-measurement using a personal lancing device and BGM. If this is not possible, a BGM should be properly cleaned and disinfected after every use according to the manufacturer's instructions. To mitigate the risk of the transmission of blood-borne pathogens, the manufacturer should specify an effective procedure for multiple cleaning and disinfection cycles that accommodates normal use over the lifetime of the BGM, which is normally considered to be 3–5 years [7, 8]. In addition, a risk that an inappropriate cleaning and disinfection procedure could lead to inaccurate readings by BGMs exists, which would make it difficult to decide upon a treatment for a patient. According to the International Organization for Standardization (ISO) standard 15197:2013 [9], manufacturers that submit a new or amended medical device license application for a BGM and/or a lancing device must include evidence of the effectiveness of the cleaning and disinfection procedure, the robustness of the device to withstand disinfection over its anticipated life cycle, labeling information about its precision, linearity, hematocrit, and its performance at different temperatures, humidities and altitudes.

After the adequate performance of a BGM is certified by a regulatory agency, studies have found that the performance of a disinfected BGM did not match the level of performance required by the regulatory agencies for the initial clearance. BGMs are regulated for accuracy to minimize errors [9–18] because information from these devices is used to make treatment decisions. However, erroneous readings can lead to incorrect treatments, which in turn can lead to excessively low blood glucose values and hypoglycemic episodes or alternatively, to inadequate lowering of blood glucose levels. Such deterioration of BGM performance could be due to an inappropriate and inadequate cleaning and disinfection procedure and the adverse effects of disinfectants [8, 19]. Specifically, most BGM systems are composed of medical thermoplastic materials including acrylonitrile butadiene styrene (ABS), polymethyl methacrylate (PMMA), or polycarbonate (PC) [19, 20]. Because some common disinfectants have been demonstrated to damage the plastic components of some meters, leading to screen failure and the breakdown of rubber control keys [19, 20], a BGM should be designed to be robust and reliable to accommodate actual use conditions [8]. In addition, point-of-care healthcare settings should implement an effective disinfection procedure according to the manufacturer's instructions for users to follow, thereby ensuring safety and efficacy [8]. A standard set of *in vitro* disinfecting approaches for evaluating the performance of commercialized BGMs under controlled conditions can facilitate clinical translation and ensure the adequate control of a patient’s blood sugar level.

ISO 15197:2013 is one of the most widely accepted standards for the assessment of the accuracy of a BGM system [9]. In ISO 15197:2013, system accuracy is stringently defined as the closeness of agreement between a measurement result and an accepted reference value determined by the manufacturer’s measurement procedure. According to this regulation, at least 95% of the system measurement results must fall within ±15 mg/dL of the results of the manufacturer’s measurement procedure at BG concentrations <100 mg/dL and within ±15% at BG concentrations of ≥100 mg/dL. Recently, the performance of newly approved BGM systems have been based on ISO 15197:2013 and verified by the device manufacturers. In addition, mandatory compliance is recommended after a 36-month transition period. Although the accuracy of a BGM system was strictly regulated in ISO 15197:2013, the physical or chemical deterioration of composed plastic components of BGM is lack of systematical and thorough investigation.

While the accuracy and reliability of subsequent generations of sensors have improved over the years, proper and clear instructions to clean BGMs can improve the reliability of readings so appropriate treatment decisions can be made [8, 9, 21]. Doctors and other healthcare providers must remind patients about how the disinfection effectiveness might affect the performance of commercially available BGM systems. In this study, the performance of 5 BGM systems was independently characterized in accordance with the accuracy criteria in ISO 15197:2013. The measurement quality of 5 BGM systems was compared in a standardized manner under controlled laboratory conditions and in practical use. A validation study with respect to HBV was systematically performed to evaluate the disinfection efficacy and system accuracy of 5 BGMs before and after disinfection according to the requirements in ISO 15197:2013.

## Materials and methods

**Fig 1:**
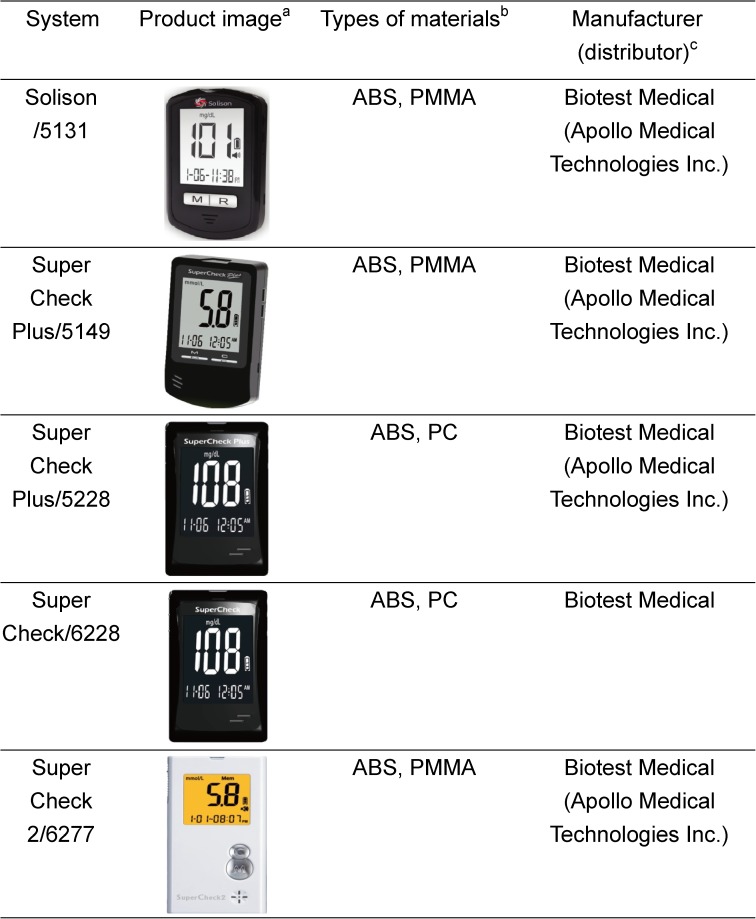
Product images and the principal materials for the 5 systems evaluated in this study. ^a^ Product images are reprinted from [http://www.btm-medical.com/en/product-list.php?id=38] under a CC BY license, with permission from the Biotest Medical Corp., original copyright 2013. ^b^ Full name of the principal types of materials: acrylonitrile butadiene styrene (ABS), polymethyl methacrylate (PMMA), polycarbonate (PC). ^c^ Distributor is given if different from manufacturer (according to the manufacturer’s labeling).

The validation of the cleaning and disinfection procedure and the clinical study of the accuracy of 5 BGMs before and after disinfection were performed between March 2014 and April 2016. The cleaning and disinfection procedure and the study of meter accuracy after cleaning and disinfection procedure in clinic were approved by the Jen-Ai Hospital Ethics Committees (IRB No. 98–02 and IRB NO. 102–07), and the appropriate authorities were notified. Informed consent forms were signed by all participants prior to the implementation of the clinical study procedures. The BGM systems for self-testing in this study are listed in Fig 1. The Solison/5131, Super Check Plus/5149, Super Check Plus/5228, Super Check/6228, and Super Check 2/6277 were provided by the Biotest Medical Corp., Taichung City, Taiwan. The Solison/5131, Super Check Plus/5149, Super Check Plus/5228, and Super Check 2/6277 were distributed by Apollo Medical Technologies Inc. and introduced to the UK market. The principal materials that composed the 5 BGMs were ABS, PMMA, and PC and were excised from 5 BGMs. The principal material samples and 5 BGMs were cleaned and disinfected using the U.S. Environmental Protection Agency (EPA)-registered disinfectant wipes (Clorox® Germicidal Wipes manufactured by Clorox Co. (EPA registered No. 67619–12)). All the cleaning and disinfection procedures were carried out in a class II biosafety cabinet (BSC). The demonstrations of multiple cleaning and disinfection procedure for Solison/5131 and Super Check Plus/5149 were bench tested in the laboratory; whereas, the Super Check Plus/5228, Super Check/6228, and Super Check 2/6277 were demonstrated in clinic. All the components and ingredients of test strips used in this study were the same except different lots (listed in Table 1). The systems displayed plasma BG values in mg/dL.

**Table 1:** Detailed information for the 5 systems evaluated in this study.

System	Test strip enzyme	Comparison method	Calibration instrument for blood glucose measure	Measurement range (mg/dL)	Measurement conditions			Test strips		
					Humidity (%)	Temperature(°C)	Hematocrit (%)	Lot 1 (No., expiry date)	Lot 2 (No., expiry date)	Lot 3 (No., expiry date)
Solison/5131	GDH-FAD	GOD	YSI 2300	20~600	20~80%	10~40°C	20~60%	S4I07026A 2016.09	S4J00908A 2016.10	S4K06405A 2016.11
Super Check Plus/5149	GDH-FAD	GOD	YSI 2300	20~600	20~80%	10~40°C	20~60%	S4I07026A 2016.09	S4J00908A 2016.10	S4K06405A 2016.11
Super Check Plus/5228	GDH-FAD	GOD	YSI 2300	20~600	20~80%	10~40°C	20~60%	S2H00106A2014.08	S2H00107A 2014.08	S2H00208A 2014.08
Super Check/6228	GOD	GOD	YSI 2300	20~600	20~80%	10~40°C	32~56%	S4G04221A2016.07	S4G04422A 2016.07	S4G05628A 2016.07
Super Check 2/6277	GOD	GOD	YSI 2300	20~600	20~80%	10~40°C	32~56%	S4G04221A2016.07	S4G04422A 2016.07	S4G05628A 2016.07

### Validation of the cleaning and disinfection procedure

Experimental procedures followed the virus disinfection test method published by the FDA [9]. The disinfection effectiveness was determined by the limit of detection (LoD) of HBV for testing according to the Clinical Laboratory and Standards Institute (CLSI) EP17-A2, Protocols for Determination of Limits of Detection and Limits of Quantitation [22]. The LoD of the Abbott ARCHITECT system HBsAg assay was 0.020 IU/mL. The minimum HBV DNA in a serum sample must be approximately 10^7^–10^8^ IU/mL; otherwise, the serum sample containing HBV could not be used as an inoculum source in the tests. The clinical HBsAg positive sera were obtained from venous blood samples from a chronically HBV-infected individual and used in this test. The initial serum concentration of the virus was 7.5 × 10^7^ IU/mL. After a 10^4^ dilution, the HBsAg values were 80.0 ± 1.23 IU/mL, and the stock concentration of HBV DNA in sera used in this study was (80.0 ± 1.23) × 10^4^ IU/mL. The three principal materials of 5 BGMs, including ABS, PMMA, and PC, were tested in this study. The samples cut from test articles were placed firmly on the bench of the BSC to inoculate with 10 μL of control or HBsAg positive sera, and then wiped with disinfectant wipes. The positive control samples were inoculated with 10 μL HBV-serum with a viral activity of 80.0 ± 1.23 IU/mL. The negative control samples were inoculated with 10 μL phosphate buffered saline (PBS) and the other samples were inoculated with clinical sera containing various concentrations of HBV, e.g., 80.0 ± 1.23, 8.1 ± 0.15, 0.8 ± 0.03, and 0.08 ± 0.004 IU/mL, respectively. All samples were incubated until the serum was thoroughly dried. The samples were vortexed with 990 μL PBS and the eluent was collected for HBsAg detection by Abbott ARCHITECT HBsAg. The viral residue of HBV on the samples of test articles was evaluated by a chemiluminescence immunoassay after inoculation and disinfection.

A complete wiping treatment included conducting wiping 10 times to assure a one minute contact time of the disinfectant as stipulated on the label of the disinfectant wipes. The treated samples were exposed to air and immediately allowed to dry, then placed in a tube containing 990 μL PBS. The tubes were vortexed for 3 minutes and 990 μL of the eluent in each tube were collected for HBsAg detection. HBsAg detection of the eluent from each test group was performed in duplicate for each serum. The HBsAg level of the eluent from each test sample should be less than 0.020 IU/mL according to a study on determination of LoD of Abbott ARCHITECT HBsAg assay system. The HBV disinfection efficacy validation study was later used to develop and implement an effective disinfection procedure for cleaning the 5 BGMs used in this study.

### Robustness of BGM after multiple disinfection cycles

The samples cut from the 5 BGM were also used to demonstrate that BGMs were robust to multiple disinfection cycles according to the effective disinfection procedure defined in this study. Electron spectroscopy for chemical analysis (ESCA) was applied to analyze the elemental composition in the untreated and disinfected samples. The ESCA spectra were acquired with a Microlab 310F (Thermo VG Scientific, East Grinstead, UK) and non-monochromatic magnesium anode X-ray at 400 W, 15 kV, and 10 mA (Kα 1253.6 eV, a concentric hemispherical capacitor analyzer). In the quantitative determination of the elemental compositions, the C 1s and O 1s core level spectra were measured and calculated from the ESCA peak area with the use of correction algorithms and relative sensitivity factors [23, 24]. After 10,000 disinfection cycles, the samples were quantitatively validated by ESCA for demonstration that that the cleaning and disinfection procedures did not result in any deterioration of chemical components of meters.

### Study population for the investigation of meter accuracy before and after disinfection

Male and female subjects (≥18 years old) with type 1 or type 2 diabetes and subjects without diabetes were included. The exclusion criteria were as follows: pregnancy or lactation, severe acute disease, and/or severe chronic disease. The subject’s medical history and medication was reviewed by a physician to determine whether any usage interfering substances had been used (e.g., acetaminophen, salicylates, ascorbic acid, dopamine) according to the manufacturer’s label.

### Evaluation procedures for BGM accuracy before and after disinfection

Five models of BGM systems were selected to assess the minimum accuracy performance of the system after a series of repeated disinfection processes defined in this study according to ISO 15197:2013, Section 6.3.3 [9]. Three meters of each BGM model were used to test the system accuracy before and after multiple cleaning and disinfection cycles. The systems were stored, used, and maintained in compliance with the manufacturer’s label. To ensure the proper function of each system, control measurements were performed daily according to the manufacturer’s labeling prior to the test procedure. The test procedure was performed by clinical personnel well trained on the limitations of the tested systems, the manufacturer’s label requirements, safety practices, and the test procedure. The test procedure was carried out in a laboratory setting at controlled room temperature (23 ± 5°C) and humidity. Each participant washed their hands with soap and water and dried them before the finger puncture and the measurement procedure were performed. The hematocrit value of each blood sample was determined to be within 32% and 56% (based on the lowest value indicated on the manufacturer’s label). Detailed information for the 5 systems evaluated in this study was listed in Table 1.

Measurements were performed on three lots of test strips and three meters for each system. ISO 15197:2013 categorizes the blood samples into different BG concentration categories [9]. The following distribution of concentration categories was used: 5% ≤50 mg/dL; 15% between >50 and 80 mg/dL; 20% between >80 and 120 mg/dL; 30% between >120 and 200 mg/dL; 15% between >200 and 300 mg/dL; 10% between >300 and 400 mg/dL; and 5% >400 mg/dL. Ten blood donors were recruited for the accuracy test. Venous blood samples were taken from each donor in lithium heparin tubes. The blood samples were divided into at least ten portions, and then spiked or glycolyzed to adequately span of the measurement range (20–600 mg/dL) to meet the requirements specified in ISO 15197:2013. Three hundred blood samples were then measured with a single test strip lot for each meter. A total of 900 samples were tested for the validation of system accuracy after multiple disinfection cycles. BG measurements were performed with five systems (three meters per system, respectively). The system accuracy of the test method was compared with the reference method, as determined by a Yellow Springs Instrument (Model YSI 2300, YSI Incorporated, Yellow Springs, OH). Both methods used for comparison provided BG values in mg/dL. Samples were assigned to a category according to the mean BG result. Residual blood was wiped off before a measurement was made and before sample collection for comparison.

### Data analyses

The system accuracy was assessed by comparing the system measurement results with the respective mean determined by the comparison measurement, which was obtained immediately before and after the measurements made with the system. At BG concentrations <100 mg/dL, the relative number of system results within ±15 mg/dL, ±10 mg/dL, and ±5 mg/dL of the comparison measurement were calculated. At BG concentrations ≥100 mg/dL, the relative number of system results within ±15%, ±10%, and ±5% of the comparison measurement were calculated. Acceptability of a system was determined by adding the number of system results within ±15 mg/dL at BG concentrations <100 mg/dL to the number of system results within ±15% at BG concentrations ≥100 mg/dL. The agreement between each system and the mean comparison result was plotted as a difference-plot as recommended in ISO 15197:2013 [8, 9]. The difference-plot showed the deviation of a single BG system measurement from the respective mean comparison measurement.

## Results

**Fig 2:**
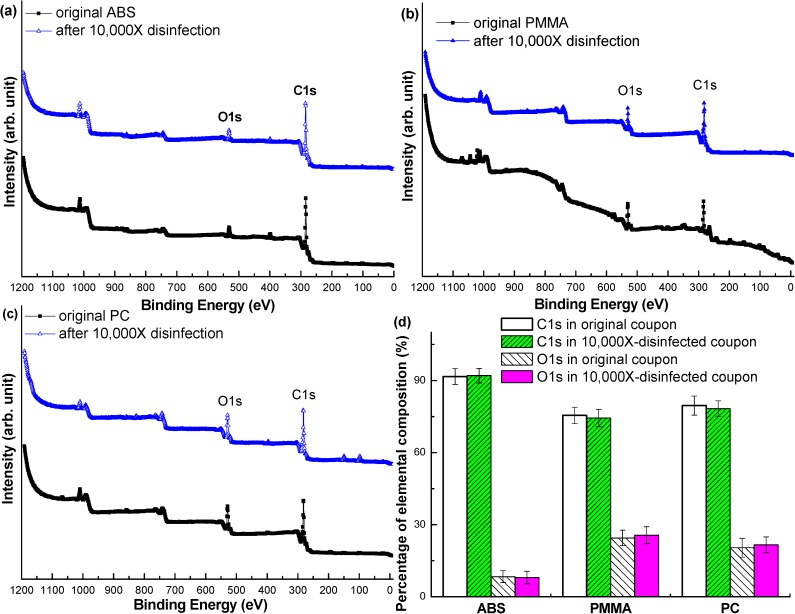
The analyses of elemental composition of the three principal materials of the 5 BGMs before and after multiple disinfection cycles were shown in (a), (b), and (c). The quantification of chemical elements before and after multiple disinfection cycles was statistically analyzed and shown in (d). Six samples of each material were tested (N = 6, value = mean ± SD.).

The cleaning and disinfection efficacy procedure in this study was used to verify the disinfection efficacy of the 5 BGM systems, the Solison/5131, Super Check Plus/5149, Super Check Plus/5228, Super Check/6228, and Super Check 2/6277. The cleaning and disinfection efficacy was determined by a LoD of HBV using the Abbott ARCHITECT HBsAg assay system. First, sera containing HBV were obtained from venous blood samples. The samples from the 5 BGM systems were then inoculated with the sera and cleaned using disinfectant wipes for 1 minute. The respective spectra of ESCA survey of ABS, PMMA, and PC in Figs 2(A)–1(C) show that the elemental compositions were not different between the initial samples and samples that were cleaned and disinfected 10,000 times. The primary elemental component of ABS was carbon, and the O 1s peaks in Fig 2(A) were related to the oxidative groups due to oxygen plasma cleaning. The C 1s were determined as approximately 92% before and after multiple disinfection cycles of the ABS samples. The principal peak areas of the C 1s and the O 1s in PMMA samples were approximately 75% and 25% before and after multiple disinfection cycles, respectively. On the other hand, the areas of the primary peaks of C 1s and O 1s in the PC samples were approximately 79% and 21% before and after multiple disinfection cycles, respectively. Briefly, the results of the quantitative elemental compositions of the tested samples were approximately the same before and after multiple cleaning and disinfection cycles, as shown in Fig 2(D).

The results of cleaning and disinfection of HBV for ABS, PMMA and PC for the disinfection efficacy of the BGM systems are listed in Table 2. HBsAg detection of the eluent from each test sample had to be less than 0.020 IU/mL per the study results of the LoD determination by the Abbott ARCHITECT HBsAg assay system. HBsAg could be detected at approximately 80 IU/mL in direct virus control after inoculation with 10 μL of donor serum, and no differences among the test articles were observed. Negative control (blank) data (PBS inoculation) did not show that any contamination occurred during our procedures. The HBsAg value after treatment with serum and disinfectant wipes for each material from one site were not greater than 0.018 IU/mL.

**Table 2:** The results of cleaning and disinfection of HBV for ABS, PMMA and PC. There were 20 samples for each test item in disinfection efficacy. Data were generated by the Abbott ARCHITECT system.

Samples	Positive Control ^a^ (IU/mL)	Negative control ^b^ (IU/mL)	Disinfection efficacy				Physical deterioration ^e^	Damage of elemental compositions ^f^	Disinfection validation
			Control of HBV concentration (IU/mL)						
			80.0±1.23	8.1±0.15	0.8±0.03	0.08±0.004			
ABS	79.5±1.52 (+) ^c^	0.002± 0.001 (-) ^d^	0.018± 0.001 (-)	0.013± 0.001 (-)	0.001± 0.001 (-)	0.009± 0.001 (-)	No	No	Accepted
PMMA	78.9±1.85 (+)	0.002± 0.001 (-)	0.017± 0.002 (-)	0.013± 0.001 (-)	0.011± 0.001 (-)	0.008± 0.001 (-)	No	No	Accepted
PC	79.2±1.67 (+)	0.001± 0.0005 (-)	0.018± 0.001 (-)	0.014± 0.002 (-)	0.012± 0.001 (-)	0.009± 0.002 (-)	No	No	Accepted

N = 20, value = mean ± SD.

^a^ Samples were inoculated with positive control sera (80.0±1.23 IU/mL) to test viral activity before disinfection.

^b^ Samples were inoculated with negative control PBS solution to test viral activity before disinfection.

^c^ The result shows HBV existed on the tested coupon according to the LoD (0.020 IU/mL).

^d^ The result shows no HBV existed on the tested coupon according to the LoD (0.020 IU/mL).

^e^ The appearance of samples was judged after 10,000-time disinfection in comparison with original samples.

^f^ The data of elemental compositions were evaluated by ESCA. The results were compared with those of original samples.

This study further determined the system accuracy and measurement reproducibility for 5 BGM systems after multiple cleaning and disinfection cycles with a standardized procedure over a clinically relevant BG concentration range. Table 3 lists detailed results of 5 tested BGMs before and after disinfection, the assessment of system accuracy of 5 BGMs was assessed according to ISO 15197:2013. The raw data and detailed results of least squares linear regression of 5 BGMs for each meter system before and after disinfection were respectively shown in Supporting Information (S1–S10 Files). The minimum system accuracy requirements of this standard were fulfilled with the three test strip lots that were evaluated. The system accuracy was assessed by comparing of the system measurement results with the mean results of a comparison measurement. All of the system accuracy results before and after disinfection were obtained from 3 replicate meters for each of the 5 BGM systems and compared with the values generated by reference YSI method.

**Table 3:** System accuracy assessment for glucose concentration according to ISO 15197:2013.

Testing conditions	Meter systems	Range of BG (mg/dL)	Linear regression of 3 BGMs		Overall result within accuracy limits (±15 mg/dL and ±15%)		% at BG concentration of					
							<100 mg/dL			≥100 mg/dL		
			R^2^	Slope (95% CI)	%	n	Percentage of values (%) within the reference values			Percentage of values (%) within the reference values		
							±15 mg/dL	±10 mg/dL	±5 mg/dL	±15%	±10%	±5%
Before multiple cleaning and disinfection cycles	Solison/5131	35–514	0.99±0.001	1.00±0.017	100.0	900/900	100.0	95.2	76.8	100.0	96.1	78.5
	Super Check Plus/5149	36–522	0.99±0.001	0.99±0.019	100.0	900/900	100.0	95.4	76.9	100.0	95.6	74.9
	Super Check Plus/5228	20–600	1.00±0.000	1.00±0.001	100.0	900/900	100.0	96.5	83.3	100.0	98.9	90.9
	Super Check/6228	35–522	0.99±0.001	1.00±0.012	100.0	900/900	100.0	93.8	71.1	100.0	92.4	72.4
	Super Check 2/6277	35–522	0.99±0.001	1.00±0.006	100.0	900/900	100.0	96.0	72.0	100.0	93.2	70.8
After multiple cleaning and disinfection cycles	Solison/5131	36–520	0.99±0.001	0.99±0.010	100.0	900/900	100.0	92.6	64.4	100.0	93.6	69.2
	Super Check Plus/5149	37–523	0.99±0.001	1.00±0.012	100.0	900/900	100.0	94.2	70.2	100.0	92.6	70.2
	Super Check Plus/5228	20–600	1.00±0.000	1.00±0.003	100.0	900/900	100.0	98.4	77.0	100.0	98.0	88.1
	Super Check/6228	36–528	0.99±0.000	1.00±0.011	100.0	900/900	100.0	91.0	70.9	100.0	92.0	69.1
	Super Check 2/6277	36–528	0.99±0.001	1.01±0.013	100.0	900/900	100.0	91.5	69.7	100.0	90.8	66.4

**Fig 3:**
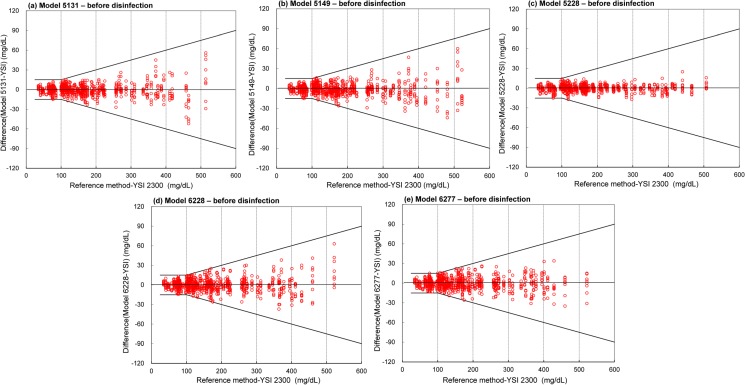
System accuracy plots for the 5 systems before disinfection. The solid lines indicate the system accuracy criteria per ISO 15197:2013. The results demonstrate that the difference between each individual glucose result falls within the acceptance limit.

Before disinfection, all 5 systems evaluated fulfilled the accuracy requirements specified by ISO 15197:2013 with at least 95% of the system measurement results within ±15 mg/dL of the results of the manufacturer’s measurement procedure at BG concentrations <100 mg/dL and within ±15% at BG concentrations ≥100 mg/dL (in Fig 3 and Table 3). The accuracy results for the Solison/5131 system showed 207 of 207 hypoglycemic samples were within ±15 mg/dL at a glucose concertation <100 mg/dL and 693 of the 693 samples with a glucose concentration of ≥100 mg/dL were within ±15% of the reference values. In the same manner, the accuracy results for the Super Check Plus/5149, Super Check Plus/5228, Super Check/6228, and Super Check 2/6277 systems independently showed 216 of 216, 198 of 198, 225 of225, and 225 of 225 hypoglycemic samples, respectively, within ±15 mg/dL at a glucose concertation <100 mg/dL. In addition, the Super Check Plus/5149, Super Check Plus/5228, Super Check/6228, and Super Check 2/6277 systems independently showed that 684 of 684, 702 of 702, 675 of 675, and 675 of 675 samples at glucose concentrations of ≥100 mg/dL were within ±15% of the reference values, respectively. Therefore, 900 of 900 samples (100%) were within the system accuracy requirements of ISO 15197:2013.

**Fig 4:**
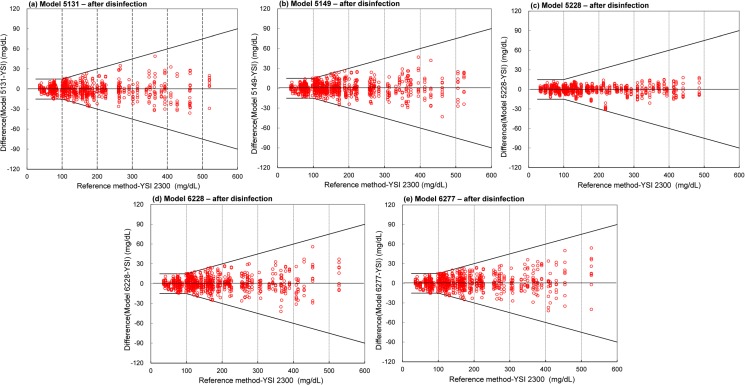
System accuracy plots for the 5 systems after disinfection. The solid lines indicate the system accuracy criteria per ISO 15197:2013. The results demonstrate that the difference between each individual glucose result falls within the acceptance limit.

Fig 4 and Table 3 show the accuracy of system measurement results after multiple cleaning and disinfection cycles within ±15 mg/dL of the results of the manufacturer’s measurement procedure at BG concentrations <100 mg/dL and within ±15% at BG concentrations ≥100 mg/dL. The accuracy results for the Solison/5131 system, Super Check Plus/5149, Super Check Plus/5228, Super Check/6228, and Super Check 2/6277 systems independently showed 216 of 216, 225 of 225, 252 of 252, 234 of 234, and 234 of 234 hypoglycemic samples were within ±15 mg/dL at a glucose concentration <100 mg/dL, respectively. In addition, the Solison/5131 system, Super Check Plus/5149, Super Check Plus/5228, Super Check/6228, and Super Check 2/6277 systems independently showed 684 of 684, 675 of 675, 648 of 648, 666 of 666, 666 of 666 samples at glucose concentrations of ≥100 mg/dL were within ±15% of the reference values, respectively. Therefore, 900 of 900 samples (100%) were within the system accuracy requirements of ISO 15197:2013.

## Discussion

Because ABS, PMMA, and PC are the most popular and common thermoplastic materials used in medical devices [20] such as BGM systems, the standard procedure for cleaning and disinfection must be adequate for a medical application. The ESCA results indicated the multiple cleaning and disinfection procedure did not deteriorate the elemental compositions of the test articles. Therefore, the cleaning and disinfection efficacy procedure in this study met the strict FDA regulations.

The virus disinfection test procedure recommended by the FDA [9] and the Abbott ARCHITECT system was successfully employed to evaluate the anti-viral residue of HBV on specific parts of the test articles in this study. The acceptance criteria for the cleaning and disinfection procedure include the infectious viral activity and deterioration of the BGMs [8]. All of the detection results were less than the LoD of each material of 0.020 IU/mL in Table 2. By the same token, the disinfection procedure was effective against HBV without causing physical and chemical deterioration in the appearance of the samples after multiple cleaning and disinfection cycles in Fig 2. Therefore, the steps of cleaning and disinfection procedure used in real BGMs are summarized as below.

**Fig 5:**
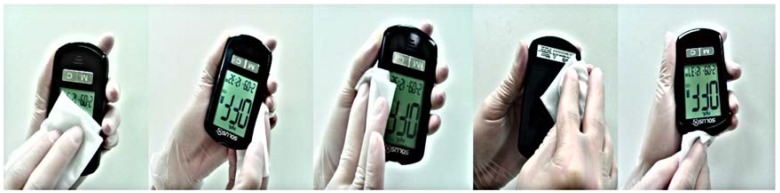
The proposed disinfection procedure for BGM cleaning. Reprinted from [http://www.btm-medical.com/en/product-list.php?id=38] under a CC BY license, with permission from the Biotest Medical Corp., original copyright 2013.

1. Clean and disinfect the 5 BGM systems, comprised of ABS, PMMA, or PC thermoplastic materials, with Clorox® Germicidal Wipes manufactured by the Clorox Co. with FPA registered No. 67619–12.
2. Put on a new pair of protective gloves before commencing the cleaning and disinfection procedure.
3. Use a Clorox wipe to clean the meter if visible dirt (e.g., food debris, grease, dust), blood or body fluids are present on the meter surface. The recommended contact time for the Clorox® Germicidal Wipes is 1 minute. This is the total time necessary for the disinfectant solution to remain on the meter surface to achieve disinfection according to the efficacy claims. Wipe down the meter as shown in Fig 5 until visible dirt or blood stains cannot be seen on the meter surface, especially on the housing, the LCD screen, the test strip port, or the buttons. If the disposable wipe is heavily saturated with disinfectant, the excess liquid should be squeezed out. Take extreme care to ensure the cleaning liquid does not enter the test strip slot or any of the meter ports.
4. Use a second disposable wipe to disinfect the meter by wiping the meter down using the method described in step 3.
5. Once the recommended contact time has elapsed, allow the meter to dry at room temperature before taking a glucose measurement from a new patient.

The systematic results and interferences of BGM accuracy have been reported in other studies [11, 14, 18, 25–27]. However, there is a lack of studies that considered the effects of cleaning and disinfection procedures on the accuracy and performance of BGMs. Inappropriate viral disinfectants may efficiently remove a contaminating infectious virus but cause the deterioration of the accuracy and damage the appearance of a BGM [19]. Our thorough and complete investigation indicates that adequate cleaning and disinfection procedures can remove infectious virus while maintaining the accuracy of a BGM. Table 3 shows that the statistical results for the 5 systems after multiple cleaning and disinfection cycles were carried out are comparable to those before multiple cleaning and disinfection cycles. The accuracy results for the 5 BGMs before and after disinfection are well within the system accuracy requirement as prescribed by ISO 15197:2013 [8, 9]. Based on our results within the required accuracy limits, the performance of 5 meters is not affected by multiple cleaning and disinfection cycles. The 5 evaluated systems before and after multiple cleaning disinfection cycles showed 100% of the measurement results were within the required accuracy limits. According to ISO 15197:2013 with a BG concentration threshold of 100 mg/dL (instead of 75 mg/dL as described in the ISO 15197:2003 standard [28]), the accuracy limit of 100 mg/dL is more stringent. In this study, before and after disinfection, all 5 systems showed 100% of the system measurement results within ±15 mg/dL of the results of the manufacturer’s measurement procedure at BG concentrations of <100 mg/dL and within ±15% at BG concentrations of ≥100 mg/dL (Table 3 and Figs 3 and 4). Our results indicate the robustness and system accuracy of the 5 BGMs after multiple cleaning and disinfection cycles according to the requirements of ISO 15197:2013 [8, 9].

Due to the risk of blood-borne pathogen transmission, it is pivotal to implement cleaning and disinfection between BGM uses [4]. The present cases of blood-borne infection are mainly associated with HBV, hepatitis C virus (HCV), and human immunodeficiency virus (HIV) [29–33]. HBV has larger pool of infectivity, high infection and environmental stability, even invisible amounts of blood or dried blood are sufficient to spread infection (in Table 4) [29–32, 34]. As a result, several incidents of outbreaks of HBV have been reported [1–6]. The disinfectant wipes (EPA registered No. 67619–12) we chose covers broad spectrum of disinfecting ability, including HBV, HIV and HCV listed in Table 4. Our step-by-step demonstration of disinfection procedure can possibly prevent other relevant blood-borne pathogen transmission among persons improperly handling or sharing BGMs. Table 4 shows the blood-borne modern epidemics and their EPA-registered disinfectants. The detail information of EPA-registered disinfectant products are available from the website: https://iaspub.epa.gov/apex/pesticides/f?p=PPLS:1. In addition, Ebola disease, one of current modern epidemics, also spreads through the direct contact with blood and body fluids of infected people. It had been proved that Ebola virus can be viable in dried blood for up to 5 days [35]. The possible transmission of Ebola virus associated with the visible or invisible dried blood should be taken into account. Although there are less EPA-registered disinfectants against Ebola virus, an economical 10% solution of household bleach, has proved no adverse effects on BGMs’ accuracy and exterior surface [19], may be an alternative. 10% bleach with our step-by-step demonstration of disinfection procedure may have beneficial effects to prevent the occurrence of transmission of Ebola virus. However, the contact time of effective disinfection on the BGM need to be further investigated.

**Table 4:** Characteristics of blood-borne modern epidemics and their EPA-registered disinfectants.

Virus	Classification	Global infection/ annual death ^a^	Transmission route ^a^	Survival in dried blood	Stability in dried blood	EPA-registered disinfectants ^b^	Ref.
HBV	DNA	2.4×10^8^/ 6.9×10^5^	Blood, body fluids	Yes	7 d	List D, List E	[29–31, 34]
HIV	RNA	3.7×10^7^/ 2.1×10^6^	Blood, body fluids	Low quantities	Several hours~ 14 d	List C, List D, List E	[29–31, 33]
HCV	RNA	1.5×10^8^/ 7×10^5^	Blood	Yes	4 d	List F	[29–31]
Ebola	RNA	2.9×10^4^/ 1.1×10^4^	Blood, body fluids	Yes	5 d	List L	[35]

^a^ Numbers are based on data published from the World Health Organisation (WHO) and the Centers for Disease Control and Prevention (CDC).

^b^ Information is based on data published from the EPA official website (https://www.epa.gov/pesticide-registration/selected-epa-registered-disinfectants).

## Conclusions

An appropriate cleaning and disinfection procedure could lead to accurate readings by BGMs exists and improve the safety of patients with diabetes. Despite measures to reduce disease transmission, a risk can occur when BGMs are used on multiple individuals or by caregivers assisting a patient. The laboratory and in-clinic performance of a BGM system before and after disinfection was demonstrated in this study to guarantee accurate readings and reliable control of BG for patients. We formulated a standard cleaning and disinfection procedure and evaluated system accuracy for the Solison/5131, Super Check Plus/5149, Super Check Plus/5228, Super Check/6228, and Super Check 2/6277 systems. All samples as well as the BGM systems showed that the cleaning and disinfection procedure was effective against HBV and did not cause any physical and chemical deterioration in the appearance of those materials. After multiple cleaning and disinfection cycles, the analytical performance of the 5 BGMs was well within the system accuracy requirement as prescribed by ISO 15197:2013. Our thorough and complete investigation demonstrated that adequate cleaning and disinfection procedures can remove HBV and maintain the accuracy of BGMs. Therefore, our validated cleaning and disinfection procedure with other EPA-registered disinfectants can be directly applied to commercial BGM products in the future.

## Supporting information

S1 File Raw data and statistic analyses of model 5131 before disinfection. (XLS)

S2 File Raw data and statistic analyses of model 5131 after disinfection. (XLS)

S3 File Raw data and statistic analyses of model 5149 before disinfection. (XLS)

S4 File Raw data and statistic analyses of model 5149 after disinfection. (XLS)

S5 File Raw data and statistic analyses of model 5228 before disinfection. (XLS)

S6 File Raw data and statistic analyses of model 5228 after disinfection. (XLS)

S7 File Raw data and statistic analyses of model 6228 before disinfection. (XLS)

S8 File Raw data and statistic analyses of model 6228 after disinfection. (XLS)

S9 File Raw data and statistic analyses of model 6277 before disinfection. (XLS)

S10 File Raw data and statistic analyses of model 6277 after disinfection. (XLS)
